# Comprehensive evaluation of community human settlement resilience and spatial characteristics based on the supply–demand mismatch between health activities and environment: a case study of downtown Shanghai, China

**DOI:** 10.1186/s12992-023-00976-z

**Published:** 2023-11-16

**Authors:** Qikang Zhong, Yue Chen, Jiale Yan

**Affiliations:** 1 School of Architecture and Art, Central South University, Changsha, 410083 China; 2 Irvine Valley College, Irvine, CA 92618 USA

**Keywords:** DPSIR model, Community human settlement resilience, Spatial heterogeneity, Geographical detector, Downtown Shanghai

## Abstract

**Introduction:**

Under globalization, human settlement has become a major risk factor affecting life. The relationship between humans and the environment is crucial for improving community resilience and coping with globalization. This study focuses on the key contradictions of community development under globalization, exploring community resilience by analyzing the mismatch between residents' health activities and the environment.

**Methods:**

Using data from Shanghai downtown, including land use, Sports app, geospatial and urban statistics, this paper constructs a comprehensive community resilience index (CRI) model based on the DPSIR model. This model enables quantitative analysis of the spatial and temporal distribution of Community Human Settlement Resilience (CR). Additionally, the paper uses geodetector and Origin software to analyze the coupling relationship between drivers and human settlement resilience.

**Results:**

i) The scores of CR showed a "slide-shaped" fluctuation difference situation; ii) The spatial pattern of CR showed a "pole-core agglomeration and radiation" type and a "ring-like agglomeration and radiation" type. iii) Distance to bus stops, average annual temperature, CO_2_ emissions, building density and number of jogging trajectories are the dominant factors affecting the resilience level of community human settlement.

**Conclusion:**

This paper contributes to the compilation of human settlement evaluation systems globally, offering insights into healthy community and city assessments worldwide. The findings can guide the creation of similar evaluation systems and provide valuable references for building healthy communities worldwide.

## Introduction

As a crucial setting for residents' daily activities, the community plays a vital role in promoting the concept of a healthy community in the era of globalization. Community human settlements are an integral part of the community system. It does not exist in isolation [1, 2]. Globalization has profoundly influenced community human settlements in various ways. Urbanization has worsened the issue of inefficient land utilization and has led to significant environmental degradation in certain regions. Simultaneously, globalization has facilitated increased international resource flows and exchanges. This can lead to resource consumption, competition and a pronounced imbalance between environmental supply and demand. Additionally, heightened cultural and social interactions may give rise to cultural conflicts and social unrest. This has negative impacts on community human settlements and human health. These factors have tangible effects on human health activities. Challenges such as resource scarcity, environmental pollution, health risks, urbanization, and social development pose significant threats to the well-being of community residents. It has become an urgent challenge to meet the needs of community residents. Particularly in countries like the United States, China, and India, large population sizes, high population densities, and frequent social mobility have led to the hollowing out and impoverishment of certain industrial and manufacturing cities, exacerbating socioeconomic inequalities. Concurrently, rapid urbanization has resulted in the gradual disappearance of urban open spaces, deteriorating urban health, and causing serious physical and mental health issues among urban residents. This has significantly impacted the connection between people and places in communities, reducing the stability and adaptability of community human settlement systems [3, 4]. Resilience, as an inherent property of systems, serves as a powerful means of maintaining stable operation and promoting sustainable system evolution. The varying levels of resilience observed in different communities directly reflect their ability to withstand diverse shocks and are closely tied to the well-being of their residents [7–9]. To address the influence of globalization on community human settlements and human health, various organizations worldwide have undertaken numerous policy initiatives. The United Nations Environment Programme (UNEP) advocates sustainable development and environmental protection to promote the sustainability of community human settlements. UN-Habitat aims to enhance the quality of the human environment in cities and communities while fostering sustainable development in the context of urbanization. Moreover, many countries have formulated policies and programs to promote the resilience and sustainability of community human settlements. Therefore, it is crucial to examine how to adapt to the risks of stress in a globalized environment and to address the tension between the health movement and environmental supply and demand. This will promote a symbiotic harmony between people and the environment and an increase in the level of resilience of community human settlements. At the same time, it will effectively improve the human settlements environment and human health.

In this context, researchers are increasingly focusing on CR [10–13]. Resilience refers to the ability of a system to maintain functional stability and adaptability in the face of shocks. Within the context of community human settlements, increased resilience can help communities effectively confront the challenges posed by globalization and safeguard the health and well-being of their residents. However, there are still certain limitations in current CR research. Firstly, the research scope has primarily concentrated on single natural ecosystems [14–18] and partially extended to relatively complex social-ecological systems [19–23]. Although these studies have emphasized optimizing and enhancing natural or social-ecological systems, they often overlook the interactions within their own internal mechanisms. Secondly, existing studies have predominantly focused on resilience concepts and indicators [24, 25], comprehensive assessments [26–29], influencing factors [30–32], and application scopes [33–35], but lack in-depth exploration of the resilience formation mechanism. Furthermore, there is a wide range of resilience assessment methods, which have evolved from qualitative studies [36–38] to a combination of qualitative and quantitative approaches such as comprehensive index methods [39, 40] and econometric methods [41, 42]. Although these findings provide methods for deconstructing CR, they tend to neglect the comprehensive impact by focusing on specific aspects. There is a two-way mutual perturbation and adaptation between residents' health activities and the environmental economy. This has important implications for the study of community resilience and community human settlements [43, 44].

In addition, improving community habitat is a priority task for achieving high-quality urban development. It can promote the sustainable development of community habitat systems and effectively alleviate the contradiction between people's aspirations for a better life and urban development [45–47]. Currently, domestic and international scholars have focused on the conceptual understanding of human settlements [46, 48] from the perspectives of sustainable development [49–51], livability [1, 52, 53], and vulnerability [54, 55]. They have employed various qualitative and quantitative methods, such as resident questionnaires [56, 57], entropy value methods [47, 58], GIS spatial analysis [50, 59], coupled coordination degree models [60, 61], and geodetector models [62], to study human settlements. These studies have explored urban human settlement systems, clarified the essence and components of human settlement systems, and identified their evolutionary trends.

In order to gain a deeper understanding of community resilience, this paper provides a comprehensive assessment of the community resilience index based on the DPSIR model from five perspectives. The paper further assesses the similarity and spatial variability of human community resilience through the spatial Moran index and ArcGIS. Finally, the paper uses a geodetector model to provide a comprehensive analysis of the factors that may have an impact on community resilience. Compared with existing articles, the innovations of this paper are as follows. First, the paper incorporates the conflict between health activities and the environment into the evaluation system of community resilience. This provides fresh insights into clarifying the meaning and formation mechanisms of community resilience. Second, the paper provides a new perspective and approach to community resilience governance in Shanghai by analyzing spatial differences and similarities. Thirdly, the paper analyses the mechanisms influencing community resilience from the perspectives of the natural environment, socioeconomics and human health. Finally, the paper provides certain strategic methods for the improvement of the resilience of different human communities. This paper provides scientific basis and practical guidance for community development and human health by constructing a sustainable and healthy community habitat system.

## Data and methods

### Overview of the study area

Shanghai is located in eastern China. It is an important economic, financial, trade, shipping, scientific, cultural and educational center. Shanghai is located in the harbour of the Yangtze River Delta region, which is an important link between China's inland and the sea. The geographical location is very important. As a cosmopolitan city, Shanghai has the world's largest container port and the second largest financial center. The famous Pudong Financial and Trade Zone and Waigaoqiao Free Trade Zone as well as China's only free trade zone are located here. Moreover, Shanghai is a globally popular tourist destination. In short, Shanghai is one of the most important cities in China. It not only has significant influence and development potential domestically, but also plays an increasingly critical role on the global stage. The pursuit of high quality has become a focal point of Shanghai's development, and the level of CR has become an important criterion for testing whether its development quality is high or not.

**Fig. 1:**
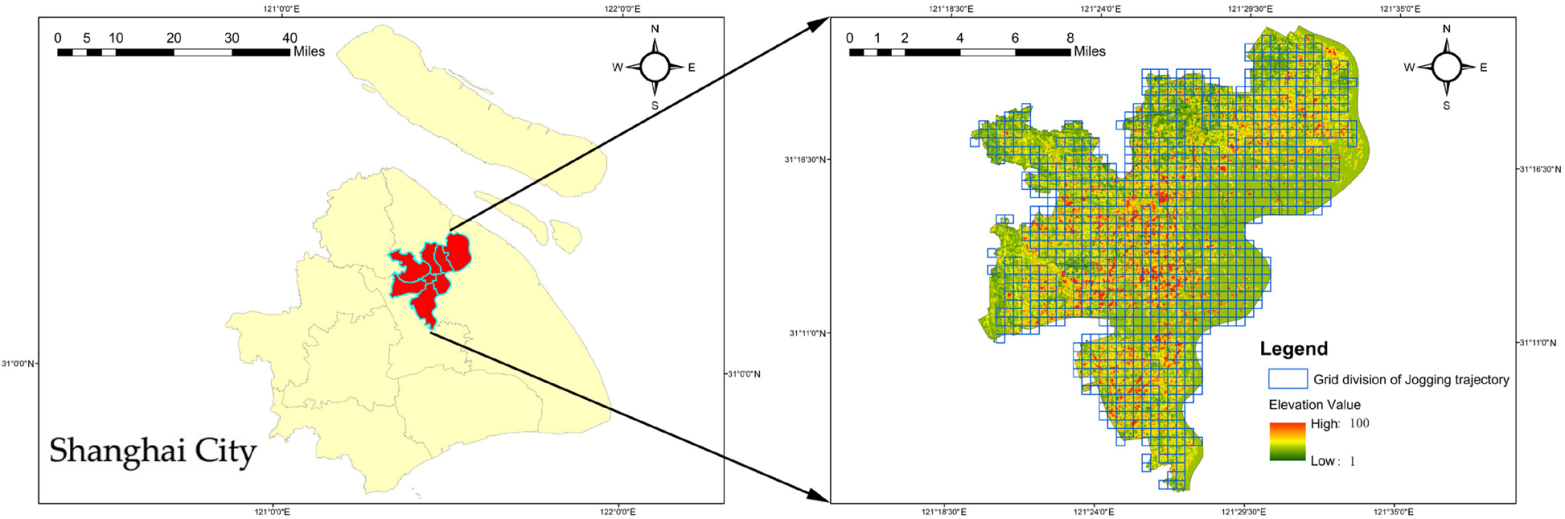
Overview of the study area

Geographically, Shanghai spans an area of 6,340.5 square kilometers and consists of 16 districts. To address the complexities of population density, urban dynamics, and health activity challenges, the paper focuses on seven central districts: Hongkou, Huangpu, Jing'an, Putuo, Xuhui, Yangpu, and Changning (as shown in Fig. 1). These districts were selected based on data accessibility and coverage, ensuring the study's reliability.

### Data subjects and sources

**Fig. 2:**
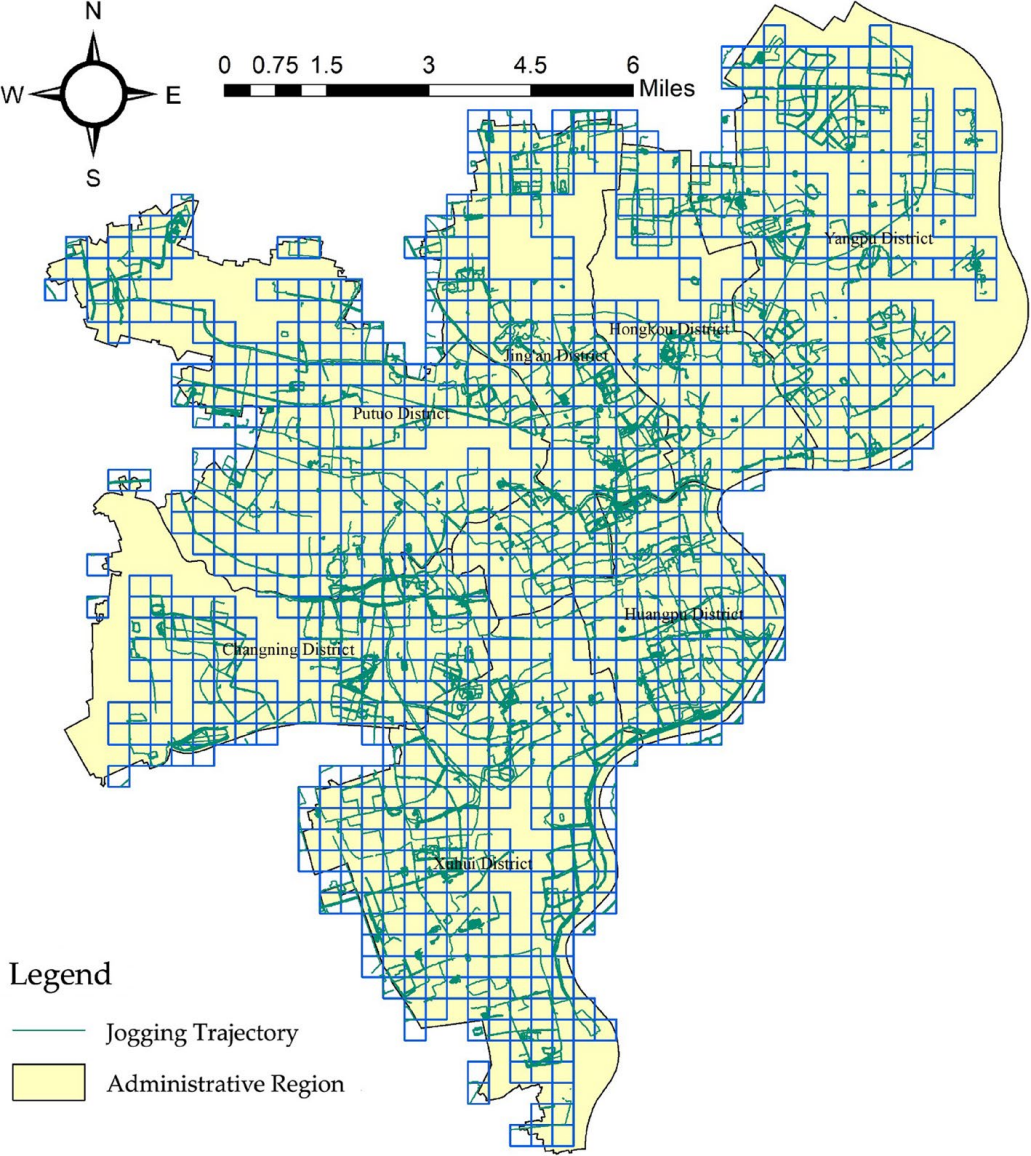
Data map of residents' jogging trajectories

The model used in this study combines data on residents' health activities, socioeconomics, ecological environment and urban geography. The data primarily originates from the Shanghai Statistical Yearbook, Shanghai regional statistical bulletins, and regional environmental status bulletins. Meteorological data is sourced from the China Air Quality Online Monitoring and Analysis Platform (https://www.aqistudy.cn/).Geospatial data is obtained from the Resource and Environmental Science Data center (http://www.resdc.cn/). To capture residents' health activity trajectories, this paper utilizes jogging data from the Dorray Sports APP (as shown in Fig. 2). All data was collected in 2018. In order to achieve a balance between computational efficiency and matching accuracy, the spatial analysis unit is set as a regular grid with a side length of 500 m. Data pre-processing includes collection, cleaning, coordinate decoding, projection conversion, and other procedures.

### DPSIR model and indicator system construction

**Fig. 3:**
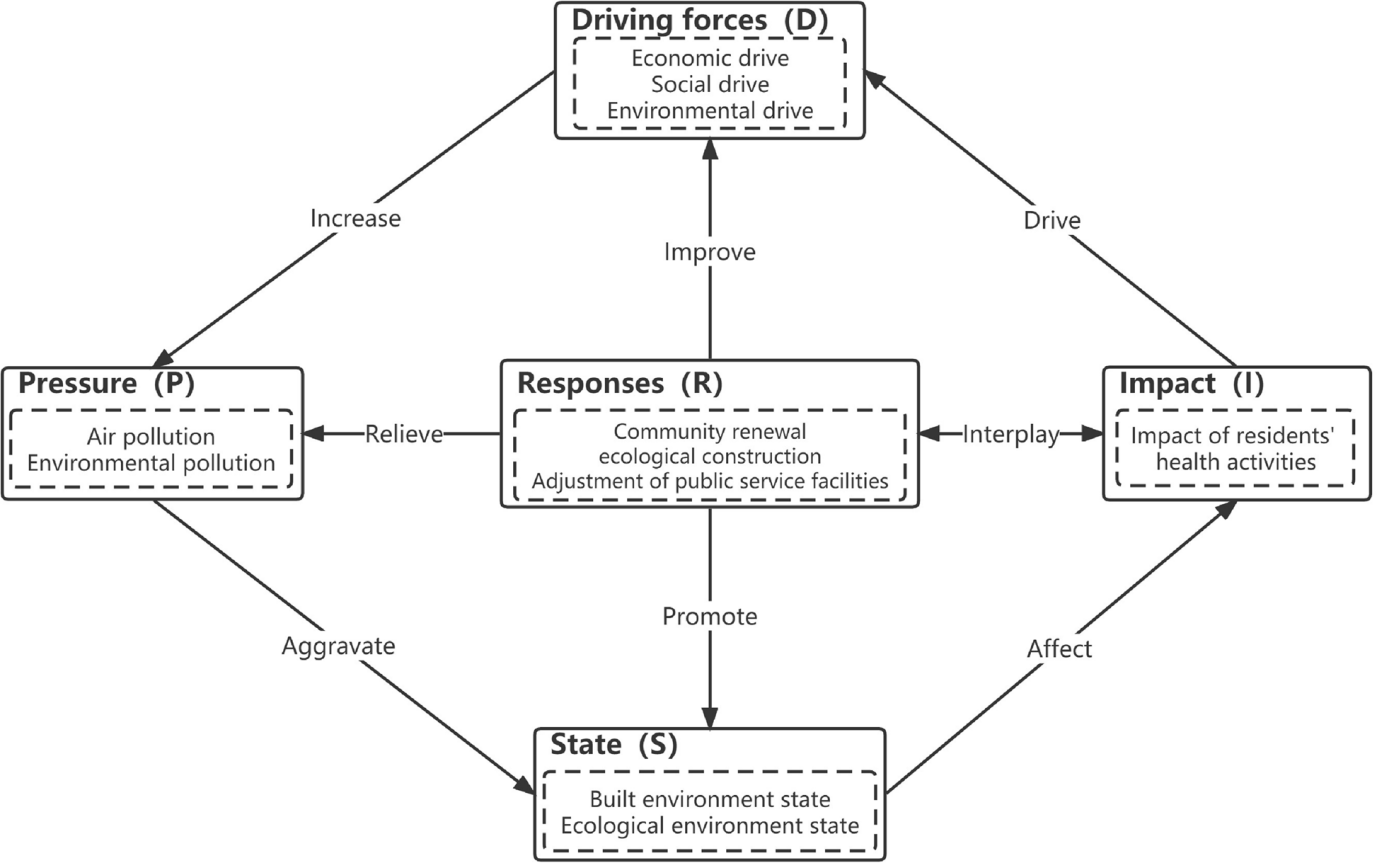
CR evaluation system DPSIR model

Figure 3 represents the DPSIR model of the CR evaluation system. The DPSIR model represents: “Driving forces (D)”, “Pressure (P)”, “State (S)”, “Impact (I)”, “Responses (R)” five dimensions respectively. It is an optimization and development of the PSR and DSR models, which can analyze the intrinsic links between activity, economic, social and environmental factors from a systemic perspective.

This study introduces the DPSIR model to assess the resilience of community human settlements. The model uses five levels and twenty-five indicators (Table 1). Within this model, drivers (D) exert pressures (P) on the environment, leading to changes in environmental states (S). This further affects human activities and health (I). Community environments respond (R) by providing feedback on drivers, pressures, states, and impacts (R) to promote healthy city development.

**Table 1:** Human settlement resilience evaluation system based on the DPSIR model

Target layer	Criterion layer	Indicator layer		Unit	Indicator attribute	Indicator layer weighting values
Human settlement resilience evaluation indicator System	Driving force (D) (0.2759)	D1	Regional Gross Domestic Product (GDP)	Billion	+	0.0206
		D2	Regional Gross National Product (GNP)	Million	+	0.0121
		D3	Population density	%	-	0.0012
		D4	Total imports and exports	Billion	+	0.0258
		D5	DEM	/	+	0.0072
		D6	Annual precipitation	L/m^2^	-	0.0106
		D7	Average annual temperature	℃	-	0.0117
	Pressure system (P) (0.0708)	P1	CO_2_ emissions	Ton	-	0.0152
		P2	PM2.5 emissions	Ton	-	0.0156
		P3	Amount of dustfall	Ton-km^2^-month	-	0.0111
		P4	Average daily volume of wet and dry waste	Ton	-	0.0238
	Status system (S) (0.0901)	S1	Building density	%	+	0.0047
		S2	Road density	%	+	0.0101
		S3	Bus stop density	%	+	0.0018
		S4	Metro density	%	+	0.0559
		S5	Density of the water system	%	+	0.1395
		S6	Land use mix	%	+	0.2355
	Impact system (I) (0.1449)	I1	Number of jogging trajectories	/	+	0.0499
		I2	Total jogging length	m	+	0.0712
	Response system (R) (0.4183)	R1	NDVI	%	+	0.0051
		R2	Green space density	%	+	0.1928
		R3	Nighttime light index	/	+	0.0063
		R4	Facility functional mix	%	+	0.0016
		R5	Distance from metro station	m	+	0.0252
		R6	Distance to bus stops	m	+	0.0455

Drawing on previous research and experimental requirements [63–78], this study identifies key indicators for each criterion level. Drivers (D) encompass dynamic elements driving resilience in community human settlements. Selected indicators include regional GDP, Regional GNP, population density, total imports and exports, DEM, and climate factors [63, 64]. They reflect economic, population, and trade dynamics. These drivers influence pressure factors, state factors, and response factors, affecting residents' health activities and the environment.

Pressure (P) refers to elements stressing the resilience of community human settlements. Indicators include CO_2_ emissions, PM2.5 emissions, amount of dust fall, and average daily volume of wet and dry waste. They reflect environmental pressures and pollution in the community [65, 66]. These factors impact state and response factors, affecting air quality and residents' health activities [67].

State (S) represents the current conditions of the health activity environment influenced by drivers and pressures. Indicators such as building, road, bus stop and metro density, density of the water system, and land-use mix reflect environmental conditions and community features [68–70]. These factors are influenced by drivers and pressures and also impact other factors and responses. For example, high building and road density may lead to congestion and transportation issues. This will be detrimental to the residents' health activities [71, 72].

Impact (I) refers to the outcomes of environmental elements on human health activities. Indicators like number of jogging trajectories and total jogging length reflect community resources for physical exercise [73–75]. These indicators directly affect residents' health activities. This is because more trajectories and longer lengths provide more opportunities for exercise and encourage resident participation [73, 74].

Response (R) indicators capture human feedback on the natural, social, and built environment and their impact on health activities. Indicators such as NDVI, green space density, nighttime lighting, facility functional mix, distance from metro station, and distance to bus stops reflect residents' responses and preferences related to health activities [76–78].

### Calculation method of human settlement resilience evaluation index

This paper uses a combination of subjective and objective weights. The hierarchical analysis method determines the subjective weights, while the entropy weight method determines the objective weights. Through the combination of these two methods, we can get the comprehensive weights. It can reflect the importance of evaluation indicators more comprehensively and accurately. After determining the weights of the indicators in the evaluation model, we used the evaluation index formula to calculate the evaluation index for each criterion layer. Subsequently, we use the corresponding formula to derive the evaluation index for the human settlements resilience. The specific calculation of indicator weights is as follows:

1) Standardization of indicator data. The following formula is used:

1 $$\begin{array}{ll}\text{Standardization of positive indicators}&{Z}_{ij}=\frac{{x}_{ij}-\mathrm{min}{x}_{ij}}{\mathrm{max}{x}_{ij}-\mathrm{min}{x}_{ij}}\end{array}$$

2 $$\begin{array}{ll}\text{Standardization of negative indicators}&{Z}_{ij}=\frac{\mathrm{max}{x}_{ij}-{x}_{ij}}{\mathrm{max}{x}_{ij}-\mathrm{min}{x}_{ij}}\end{array}$$

where ${x}_{ij}$ is the standardised value of the $j$ th indicator for the $i$ th evaluation sample.

2) Entropy weighting method to calculate indicator weights, the formula is:

3 $$\begin{array}{ll}\text{Calculate the weight of the}\,j\text{th indicator of the}\,i\text{th evaluation sample}&{Y_{ij}} = {\raise0.7ex\hbox{${{Z_{ij}}}$} \!\mathord{\left/ {\vphantom {{{Z_{ij}}} {\sum {{Z_{ij}}} }}}\right.\kern-0pt}\!\lower0.7ex\hbox{${\sum {{Z_{ij}}} }$}}\end{array}$$

4 $$\begin{array}{ll}\text{Calculate the information entropy value of the}\,j\text{th indicator}&{{\text{e}}_{\text{j}}} = - k\sum\limits_{i = 1}^m {{Y_{ij}}\ln {Y_{ij}}}\end{array}$$

5 $$\begin{array}{ll}\text{Calculate the information entropy redundancy of the}\,j\text{th indicator}&{{\text{g}}_i} = 1 - {e_j}\end{array}$$

6 $$\begin{array}{ll}\text{Calculate the weight of the}\,j\text{th indicator}&{{\text{w}}_j} = {\raise0.7ex\hbox{${g_j}$} \!\mathord{\left/ {\vphantom {{g_j} {\sum\limits_{j = 1}^n {g_j} }}}\right.\kern-0pt}\!\lower0.7ex\hbox{${\sum\limits_{j = 1}^n {g_j} }$}}\end{array}$$

where ${Y}_{ij}$ denotes the weight value of the $j$ th indicator for the $i$ th evaluation sample.${e}_{j}$ denotes the information entropy value of the $j$ th indicator;${g}_{j}$ denotes the information entropy redundancy of the $j$ th indicator. ${W}_{j}$ denotes the value of the weight coefficient of the $j$ th indicator.

3) Hierarchical analysis method to calculate the indicator weights at the criterion level, the formula is:

7 $$B=\left(\begin{array}{ccc}{a}_{11}& \dots & {a}_{1n}\\ \dots & \dots & \dots \\ {a}_{j1}& \dots & {a}_{jn}\end{array}\right)$$

8 $${M}_{j}=\sqrt[n]{{a}_{j1}{a}_{j2}{a}_{j3}\cdots {a}_{jn}}$$

9 $${Q}_{j}=\frac{{M}_{ij}}{\sum_{j=1}^{n}{M}_{j}}\left(\mathrm{j }= 1, 2,...,\mathrm{ n}\right)$$

10 $${\uplambda }_{\mathrm{max}}=\frac{1}{n}\sum_{j=1}^{n}\frac{{a}_{jn}{Q}_{j}}{{Q}_{j}}(\mathrm{j }= 1, 2,...,\mathrm{ n})$$

11 $$\mathrm{CI}=\left({\uplambda }_{\mathrm{max}}-\mathrm{n}\right)/\left(\mathrm{n}-1\right)$$

12 $$\mathrm{RI}=\frac{{\mathrm{CI}}_{1}+{\mathrm{CI}}_{2}+\cdots +{\mathrm{CI}}_{\mathrm{n}}}{\mathrm{n}}$$

13 $$\mathrm{CR}=\frac{\mathrm{CI}}{\mathrm{RI}}$$

where ${a}_{jn}$ denotes Criterion layer indicator data; $B$ denotes the judgment matrix. ${M}_{j}$ is the geometric mean of the row vector elements of the judgment matrix; $n$ denotes the number of indicators; ${Q}_{j}$ is the weight of the jth evaluation indicator; ${\uplambda }_{max}$ is the maximum characteristic root; $CI$ is the consistency indicator; $RI$ is the random consistency indicator.

4) Calculate the human settlement resilience evaluation index. The formula for calculating the weights is:

14 $$D=\sum {W}_{Dj}{Z}_{Dj};\;P=\sum {W}_{Pj}{Z}_{Pj};\;S=\sum {W}_{Sj}{Z}_{Sj};\;I=\sum {W}_{Ij}{Z}_{Ij};\;R=\sum {W}_{Rj}{Z}_{Rj}$$

15 $$U=D\times {W}_{D}+P\times {W}_{P}+S\times {W}_{S}+I\times {W}_{I}+R\times {W}_{R}$$

where U is the human settlement resilience evaluation index. Combined with the index setting of the DPSIR model. D denotes the driving force index and ${W}_{Dj}$ is the weight corresponding to the jth indicator under the D criterion layer and ${Z}_{Dj}$ is the standardised value of the $j$ th indicator under the D criterion layer and ${W}_{D}$ is the weight corresponding to the D quasi-lateral layer; P, S, I and R index are set and calculated in the same way as the D index.

### ArcGIS spatial analysis

In the analysis process, we used the natural break method with the help of ArcGIS software to classify the CR class types based on CRI (Table 2). This can show the spatial changes of CR index in downtown Shanghai more intuitively.

**Table 2:** Classifying criteria of human settlement resilience level

District-level Human Settlement Resilience Index	CRI	Human Settlement Resilience Level
0.00—1.52	0.0078—0.0199	Very low quality
1.53–2.56	0.0200—0.0289	Low quality
2.57—3.22	0.0290—0.0411	Medium quality
3.23—4.23	0.0412—0.0608	High quality
4.24–6.62	0.0609—0.1128	Very high quality

The study used two spatial autocorrelation statistics, Global Moran's I and Local Moran's I, in the testing phase. The purpose of the methodology is to analyze the overall spatial autocorrelation characteristics and the local spatial autocorrelation characteristics of the human settlements resilience of the region.

1) Calculate the global Moran index. Where ${G}_{0}=\sum_{i=1}^{n}\sum_{j=1}^{n}{w}_{ij}$, n is the total number of spatial cells, the ${y}_{i}$ and ${y}_{j}$ denote the attribute values of the $i$ th spatial unit and the $j$ th spatial unit respectively and $\overline{y}$ is the mean value of the attribute values of all spatial units and ${w}_{ij}$ is the average value of the attribute values of all spatial units, and is the spatial weight value. The calculation formula is:

16 $$I=\frac{n}{{G}_{0}}\times \frac{\sum_{i=1}^{n}\sum_{j=1}^{n}{w}_{ij}({y}_{i}-\overline{y})({y}_{j}-\overline{y})}{\sum_{i=1}^{n}{({y}_{i}-\overline{y})}^{2}}$$

Calculate the local Moran index. where ${F}_{i}={y}_{i}-\overline{y}$,${F}_{j}={y}_{j}-\overline{y}$,${G}^{2}=\frac{1}{\mathrm{n}}\sum {({y}_{j}-\overline{y})}^{2}$,${w}_{ij}$ are the spatial weight values, n is the total number of all regions on the study area and ${I}_{i}$ then represents the local Moran index for the ith region. The formula is calculated as:

17 $${I}_{i}=\frac{{F}_{i}}{{G}_{2}}\sum_{j\ne i}^{n}{w}_{ij}{F}_{j}$$

### Geodetector model

Geodetector represents a statistical model extensively employed in the fields of geography and environmental science. It is used to examine the effects of various geographical factors, such as landforms, soils, and climate, on a specific phenomenon, including vegetation distribution, species occurrence, and land-use changes. This model dissects the phenomenon into a combination of geographical elements and quantifies the impact of each element on the phenomenon, thereby unraveling the intricate interplay between geographical factors and the observed phenomenon. Based on the analysis of spatial variation of geographical layers, this study uses factor detector to analyze the influence of internal driving factor X of five criteria layers on Y, i.e. CR. Interaction detection is used to identify the relationship among the factors that affect the resilience level of community human settlement [79, 80]. The model is as follows:

18 $$q=1-\frac{1}{N{\sigma }^{2}}{\sum }_{h-1}^{L}{N}_{h}{\sigma }_{h}^{2}$$

where: $q$ is the influence of the influence factor on the resilience of community human settlement; $h$ represents the stratification of factor X. ${N}_{h}$ and $N$ represent the number of cells in stratum $h$ and the whole area, respectively; ${\sigma }_{h}^{2}$ and ${\sigma }^{2}$ represent the variance of Y values in stratum $h$ and the whole area, respectively. The value range of $q$ is [0, 1], with larger values indicating stronger explanatory power of factor X on attribute Y and vice versa.

## Results

### Condition of regional human settlement resilience level

Based on the resilience index, the paper ranks the resilience level of the human environment in each district in Shanghai (Table 3). Xuhui District obtained the highest resilience index score of 6.62, which is much higher than the other six districts. This indicates that its resilience quality is very high. Putuo District and Yangpu District followed closely with resilience index scores of 4.23 and 4.01. This indicates that those two districts also have a high quality of resilience. Changning District and Jing'an District obtained resilience index scores of 3.22 and 3.05, respectively, indicating a medium quality of resilience. Huangpu District has a low resilience index of 2.56 but has the potential to improve towards medium quality. Hongkou District had the lowest resilience index score of 1.52, indicating a very low quality of resilience. The resilience index of Hongkou District is significantly different from the other districts.

**Table 3:** Regional-scale human settlement resilience rating index ranking

District name	CRI	Ranking
Xuhui District	6.62	1
Putuo District	4.23	2
Yangpu District	4.01	3
Changning District	3.22	4
Jing'an District	3.05	5
Huangpu District	2.56	6
Hongkou District	1.52	7

**Fig. 4:**
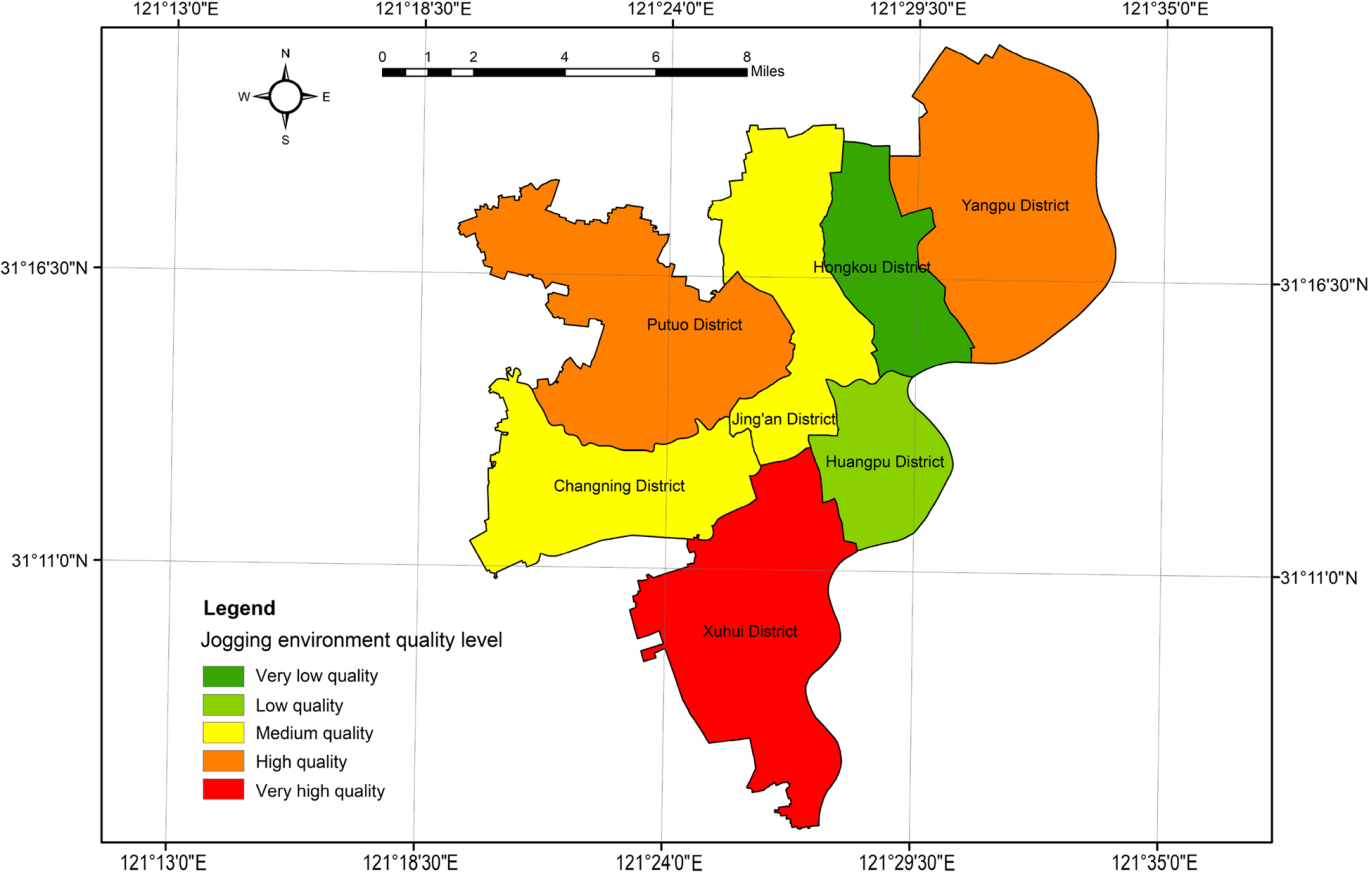
Spatial distribution of regional-scale human settlement resilience level

Regarding the spatial distribution (Fig. 4), the overall pattern in the basin displayed a central area with lower resilience levels and a peripheral area with higher resilience levels. Xuhui District stood out as relatively high in resilience, followed by Putuo District and Yangpu District.

### Condition of CR level

**Fig. 5:**
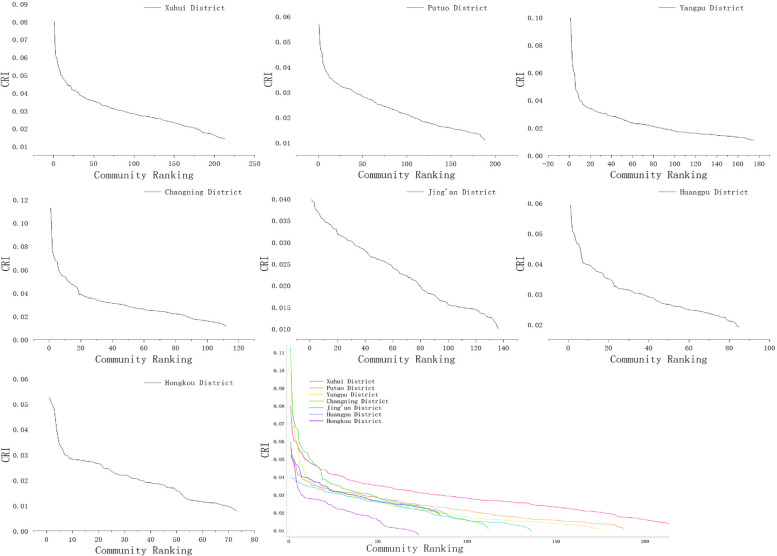
Analysis of community-scale human settlement resilience Level by district

Based on Fig. 5, we rank the CRI of each district from highest to lowest. It was observed that Xuhui and Putuo districts exhibited a gradual decline in the CRI, with relatively smooth curves. The CRI for Yangpu District initially shows a sharp downward trend, followed by a more stable downward trend. It eventually fluctuates slightly after it reaches a certain level. The downward trend of CRI in Changning District is similar to that of Xuhui District, but with some irregular fluctuations at the bend. Jing'an District displayed a linear and consistent decline in the CRI. Huangpu District initially had an irregular curve, but later exhibited a linear and steady decline after surpassing a specific threshold. Hongkou District experienced a drastic and irregular decline in the CRI.

Overall, Xuhui District displayed the highest level of community resilience compared to the other six districts. Putuo District, Yangpu District, Jing'an District, Huangpu District, and Changning District maintained a relatively consistent level of community resilience. They differ in that Changning District has a higher level of human settlements resilience. In contrast, Hongkou District had significantly lower community resilience compared to the other district. This suggests that effective measures need to be taken to improve the resilience of the district.

### Condition of high human settlement resilience communities by district

**Fig. 6:**
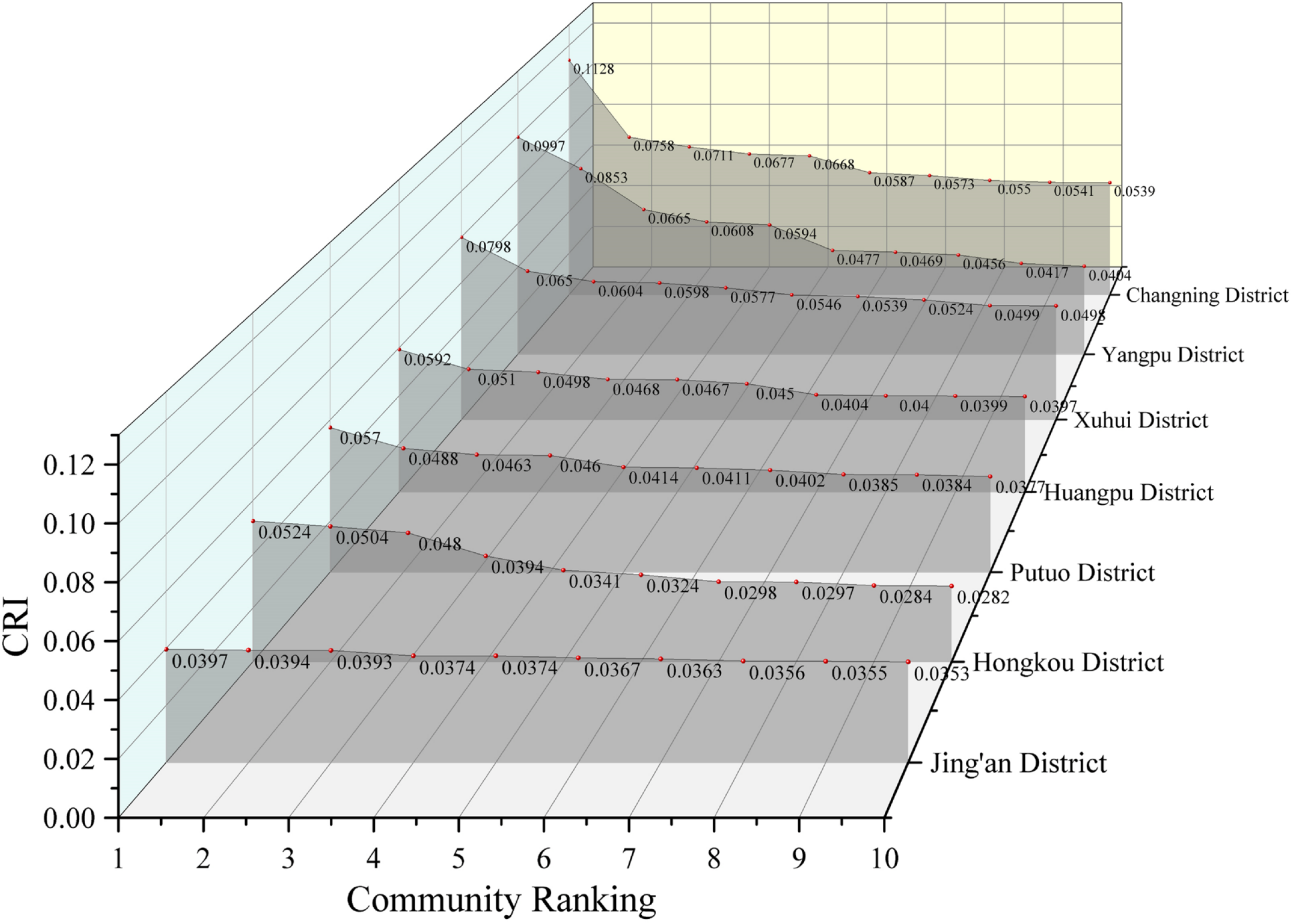
Comparison of high-quality CR Level

To gain a better understanding of communities with high human settlement resilience in each district, this study compared the top ten communities in each district (as shown in Table 4 and Fig. 6). The analysis reveals that Changning District exhibits the highest level of community resilience among the seven districts, showcasing its superior performance. Xuhui District demonstrates an overall high and consistent level of community resilience. Yangpu District also has a high level of community resilience, but there are significant differences within its range. The highest index reaches 0.0997, while the tenth ranked community scores only 0. 0404.The difference between the two is 0.0593.

**Table 4:** Regional ranking of high-quality CR level (top 10)

Ranking	Xuhui District	Putuo District	Yangpu District	Changning District	Jing'an District	Huangpu District	Hongkou District
1	0.0798	0.0570	0.0997	0.1128	0.0397	0.0592	0.0524
2	0.0650	0.0488	0.0853	0.0758	0.0394	0.0510	0.0504
3	0.0604	0.0463	0.0665	0.0711	0.0393	0.0498	0.0480
4	0.0598	0.0460	0.0608	0.0677	0.0374	0.0468	0.0394
5	0.0577	0.0414	0.0594	0.0668	0.0374	0.0467	0.0341
6	0.0546	0.0411	0.0477	0.0587	0.0367	0.0450	0.0324
7	0.0539	0.0402	0.0469	0.0573	0.0363	0.0404	0.0298
8	0.0524	0.0385	0.0456	0.0550	0.0356	0.0400	0.0297
9	0.0499	0.0384	0.0417	0.0541	0.0355	0.0399	0.0284
10	0.0498	0.0377	0.0404	0.0539	0.0353	0.0397	0.0282

The CRI values for Huangpu and Putuo districts range from 0.3 to 0.6. The values are stable but not outstanding. Only three communities in the Hongkou district have CRI values around 0.05. Both Hongkou District and Jing'an District generally display low CRI levels, ranging from 0.2 to 0.4. However, the top ten communities in Jing'an District show a tendency towards consistent CRI values. This indicates a relatively equitable development of resilience levels in the district.

### Spatial characteristics of human settlement resilience

#### CR spatial autocorrelation

**Fig. 7:**
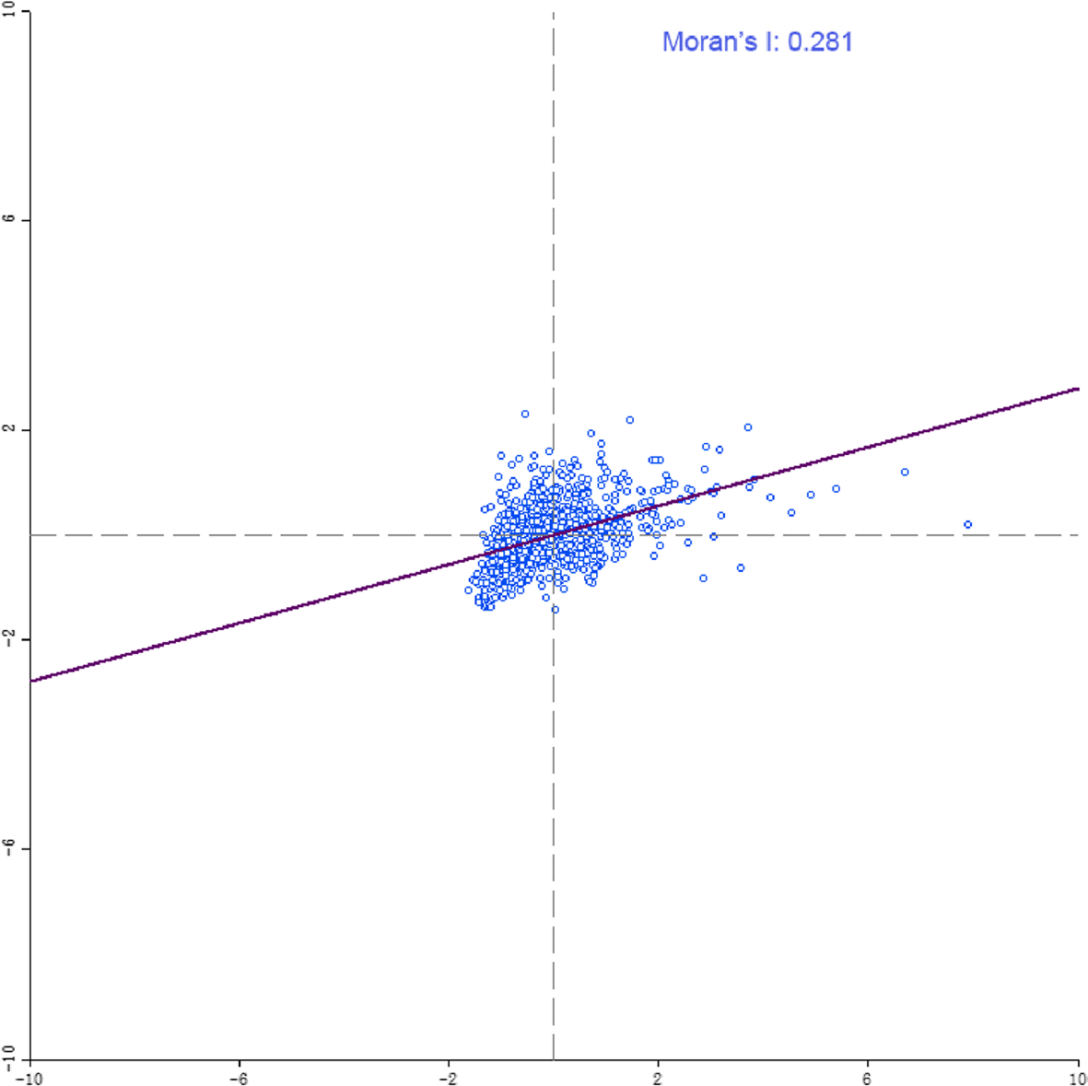
Scatterplot of Moran's I index of CR level by district in Shanghai

Based on the CRI, this study implemented a global spatial autocorrelation analysis with the help of Space/Univariate Moran's I in Geoda software. The results show that Moran's I value is 0.281. This indicates that there is a significant positive spatial autocorrelation for CRI, which exhibits spatial clustering (Fig. 7).

**Fig. 8:**
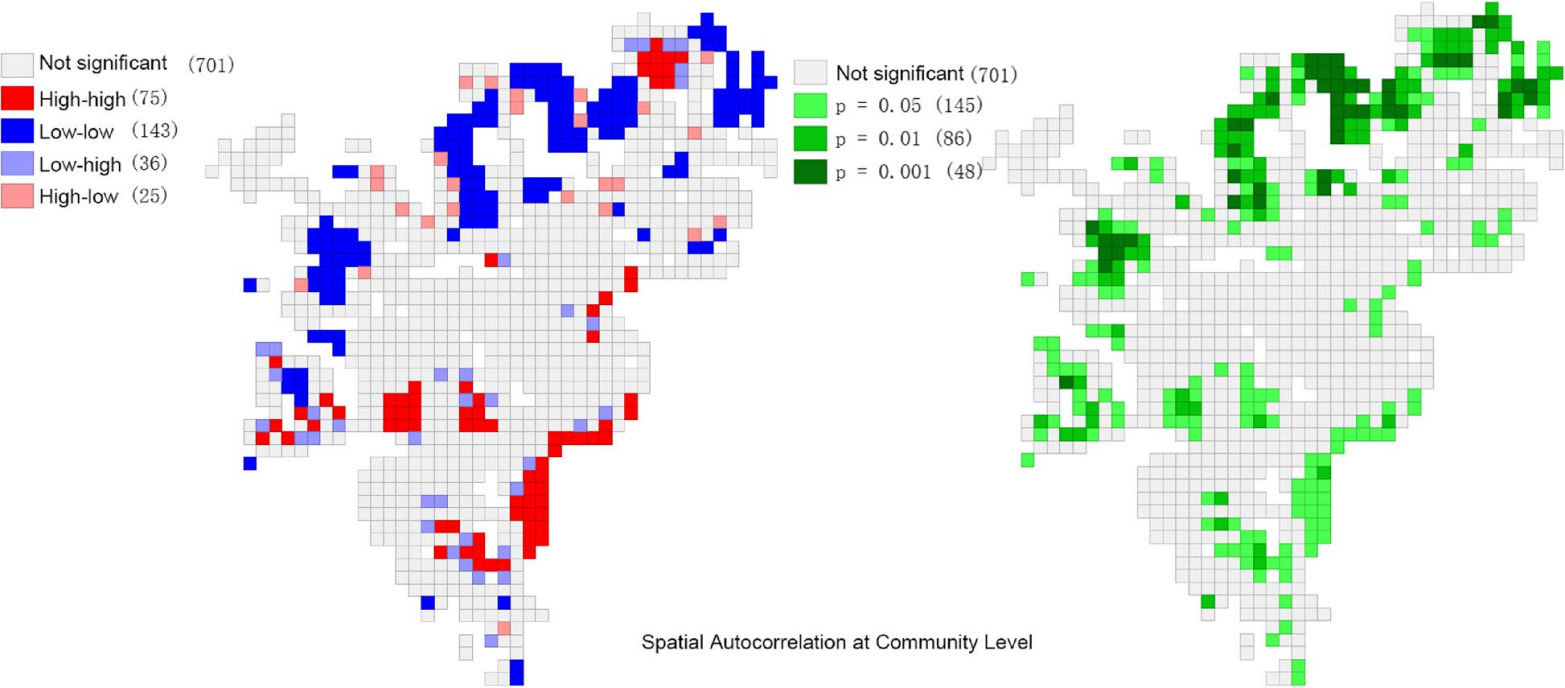
LISA chart of Moran's I index of CR level by district in Shanghai

To assess the local spatial aggregation and analyze the similarity and spatial divergence of human settlement resilience levels among neighboring community units, we used ArcGIS 10.2. As shown in Fig. 8, we have mapped the evolution of the aggregation of human settlements resilience levels across regional communities. The Moran's I index LISA plot in the "High-High" (HH) and "Low-Low" (LL) quadrants indicate a strong positive spatial correlation of CR levels. This suggests a homogeneous and aggregated distribution pattern across regions. Conversely, the "High-Low" (HL) and "Low–High" (LH) quadrants represent areas with a strong negative spatial correlation. This suggests spatial heterogeneity and discrete distribution patterns of CR levels across regions during the study period. The distribution of communities across the HH, LL, HL, and LH quadrants can be observed in Fig. 8. Communities are more concentrated in the HH and LL quadrants than in the HL and LH quadrants. Additionally, there is a notable regional concentration of overall CR levels. Communities in the LL quadrant are concentrated in the north, while those in the HH quadrant are located mainly in the south. Communities in the HL and LH quadrants are scattered around communities in the HH and LL quadrants. Overall, there is a clear spatial dependence between CR levels in each region. This indicates clustering characteristics with neighboring regions. Within the study area, fewer gathering areas are displaying a "high-low" polarization effect and a "low–high" transitional type at a significance level of 0.05.

#### Spatially descriptive statistics of CR by district

**Fig. 9:**
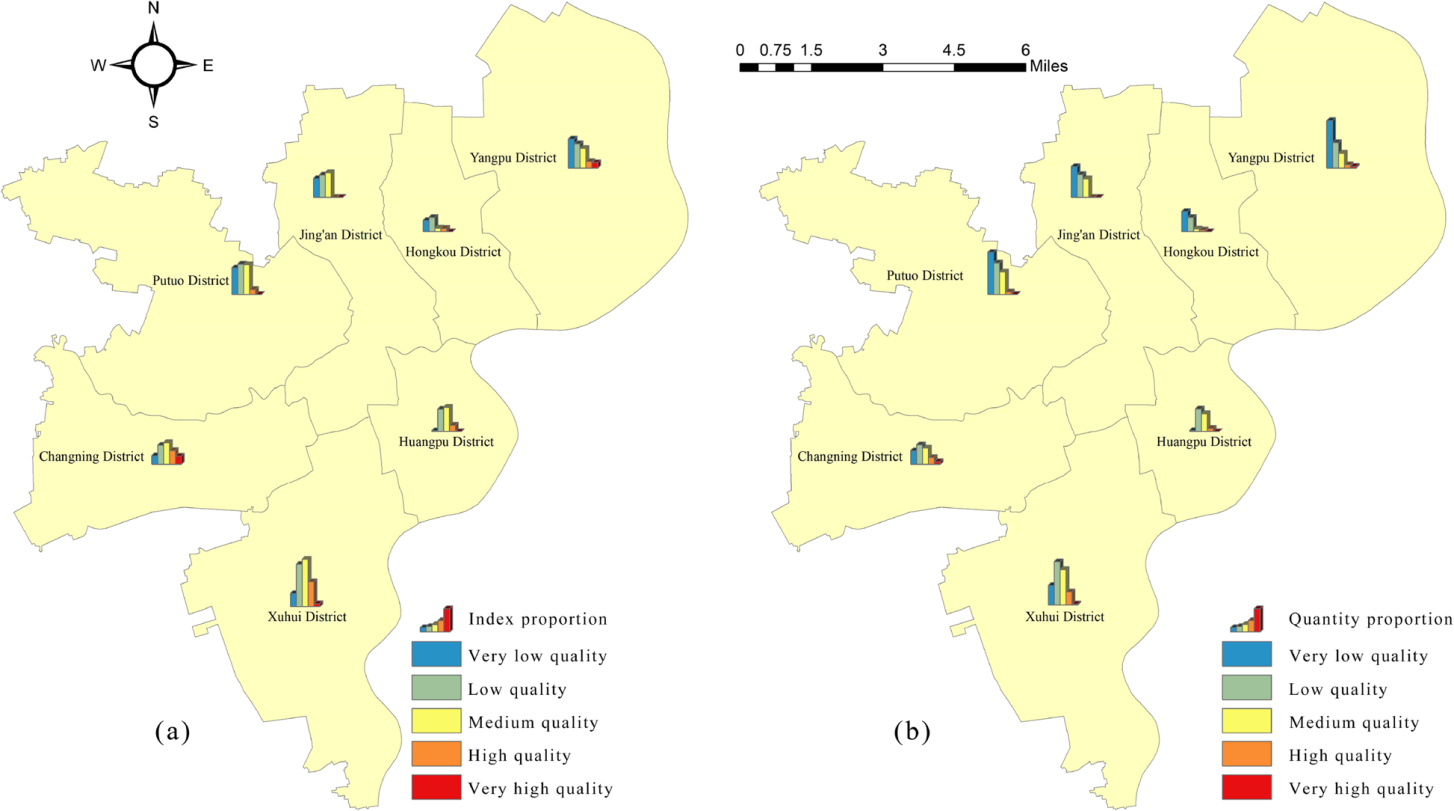
Spatial distribution proportion of communities with different human settlement resilience level in Shanghai

Based on the research formula, we calculated the human settlements resilience index for each area and derived the CRI. We then classified the CRIs into five categories: very low quality zone, low quality zone, medium quality zone, high quality zone, and very high quality zone using the natural interruption point grading method. The classification and quantity of human settlement resilience are presented in Tables 5 and 6, and the spatial distribution of the proportion of communities in different grades in the participating cities is illustrated in Fig. 9.

**Table 5:** Overall statistics for the human settlement resilience evaluation

Resilience grading	Index	Proportion/%	Number of communities/EA	Percentage of communities/%
Very low quality	5.141	20.40%	331	33.71%
Low quality	8.309	32.97%	340	34.62%
Medium quality	8.119	32.21%	243	24.75%
High quality	2.844	11.28%	58	5.91%
Very high quality	0.791	3.14%	10	1.02%

**Table 6:** CR Evaluation statistics

	**Hongkou District**		**Huangpu District**		**Jing'an District**		**Putuo District**	
	Index/%	Quantity/%	Index/%	Quantity/%	Index/%	Quantity/%	Index/%	Quantity/%
Very low quality	0.531 (35.0%)	38 (52.1%)	0.039 (1.5%)	2 (2.4%)	0.889 (28.6%)	58 (42.6%)	1.268 (29.0%)	80 (42.6%)
Low quality	0.67 (44.2%)	27 (37.0%)	1.062 (41.5%)	43 (50.6%)	1.06 (34.1%)	43 (31.6%)	1.442 (33.0%)	60 (31.9%)
Medium quality	0.165 (10.9%)	5 (6.8%)	1.156 (45.2%)	34 (40.0%)	1.164 (37.4%)	35 (25.7%)	1.418 (32.5%)	43 (22.9%)
High quality	0.151 (10.0%)	3 (4.1%)	0.299 (11.7%)	6 (7.1%)	0 (0.0%)	0 (0.0%)	0.24 (5.5%)	5 (2.7%)
Very high quality	0 (0.0%)	0 (0.0%)	0 (0.0%)	0 (0.0%)	0 (0.0%)	0 (0.0%)	0 (0.0%)	0 (0.0%)
	**Xuhui District**		**Yangpu District**		**Changning District**			
	Index/%	Quantity/%	Index/%	Quantity/%	Index/%	Quantity/%		
Very low quality	0.623 (10.0%)	37 (17.4%)	1.378 (34.3%)	90 (51.4%)	0.413 (12.1%)	26 (23.2%)		
Low quality	2.018 (32.4%)	82 (38.5%)	1.151 (28.7%)	48 (27.4%)	0.906 (26.6%)	37 (33.0%)		
Medium quality	2.256 (36.2%)	67 (31.5%)	0.93 (23.2%)	28 (16.0%)	1.031 (30.3%)	31 (27.7%)		
High quality	1.193 (19.1%)	25 (11.7%)	0.302 (7.5%)	6 (3.4%)	0.66 (19.4%)	13 (11.6%)		
Very high quality	0.145 (2.3%)	2 (0.9%)	0.252 (6.3%)	3 (1.7%)	0.394 (11.6%)	5 (4.5%)

Table 5 reveals that low quality areas have the highest proportion of CR evaluations, with a total of 340, accounting for 34.62% of the total. Very low quality areas follow with 331 evaluations, accounting for 33.71% of the total. Medium quality areas have 243 evaluations, accounting for 24.75% of the total. On the other hand, the number of very high quality and high quality zones is the smallest, with 58 and 10 zones respectively, accounting for only 5.91% and 1.02% of the total.

Table 6 and Fig. 9 provide insights into the composition of different levels of communities in the participating cities. There are obvious regional differences in the level of community building in Shanghai, with an overall distribution pattern of "northwest to southeast bulge". The distribution of the index and the number of communities exhibit similar patterns. Generally, the southeastern part of the city demonstrates higher CR levels compared to the northwestern part. Xuhui District and Changning District have the highest proportion of very high and high quality communities, accounting for 21.4% and 31% respectively, serving as the leading "twin cores" in the development of Shanghai's CR quality. Jing'an District lacks very high and high quality communities, indicating a low level of development and emphasizing the need to accelerate the construction of high-quality human settlement resilience communities. Yangpu District and Putuo District have the highest percentage of very low and low quality communities, with 78.8% and 74.5% respectively, indicating a majority of communities with low quality. The government should prioritize the development of resilient qualities in community human settlement and solve the problems of "Weak Communities" and " Fragile Communities". The government should also focus on transforming "backward communities" into "resilient communities" as soon as possible.

#### Spatial heterogeneity of CR

**Fig. 10:**
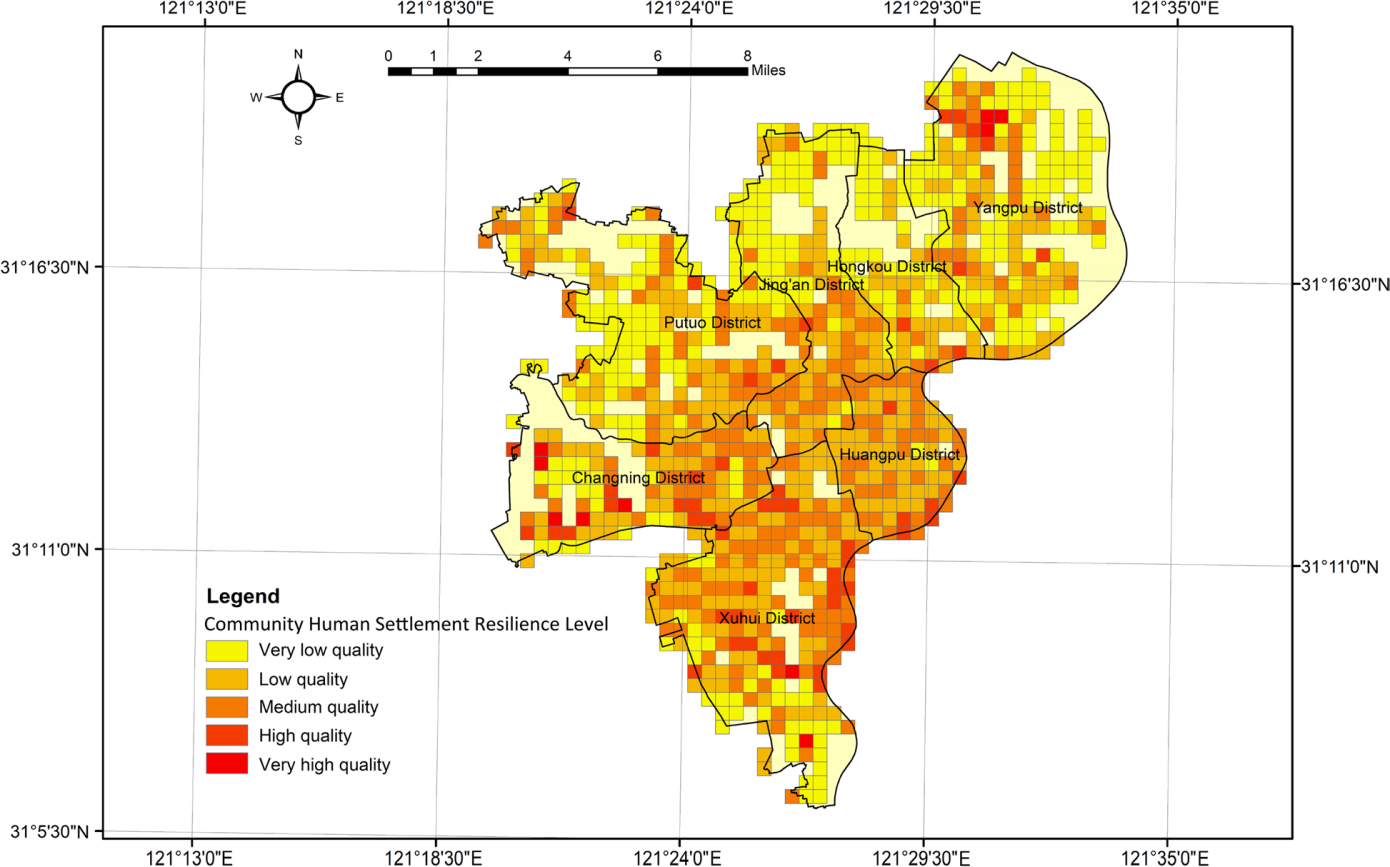
Comprehensive CR evaluation for spatial heterogeneity characteristics

**Fig. 11:**
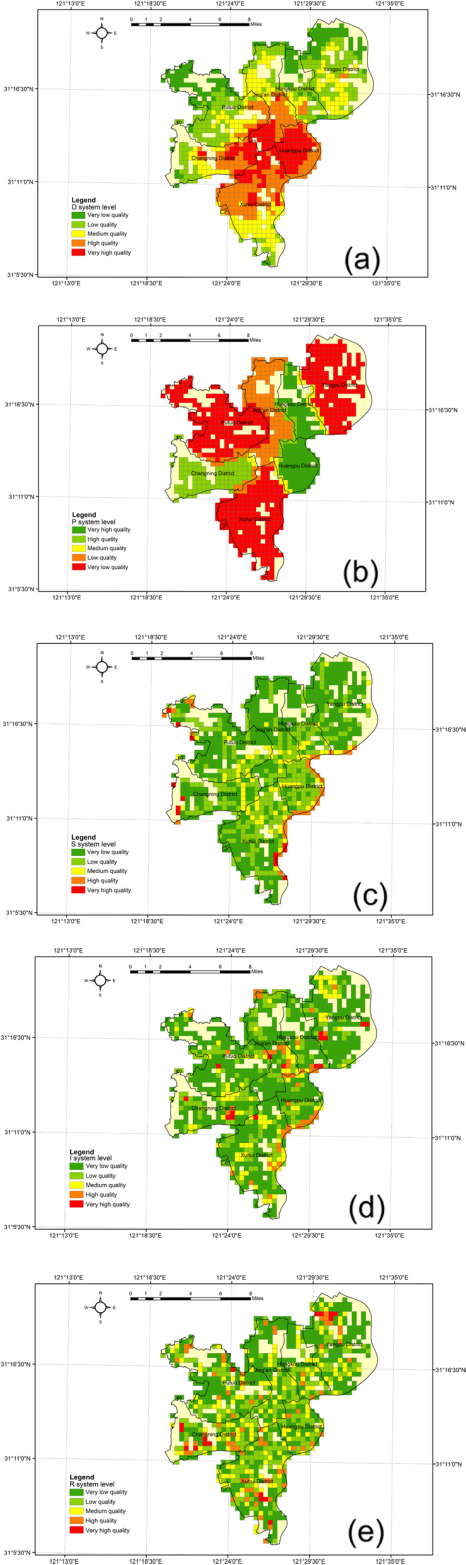
CR evaluation for spatial heterogeneity characteristics of each system

To visualize the spatial characteristics of the CR level in each region of Shanghai, we utilized ArcGIS 10.2 software to generate Figs. 10 and 11. The figures illustrate the spatial distribution of the CR level and subsystem indices.

Figure 10 reveals clear spatial differentiation characteristics of CR levels. Communities with very high human settlement resilience exhibit a "pole-core" spatial pattern, clustering around the central areas of the district and the vicinity of the Huangpu River. Core communities with medium and high human settlement resilience display an irregular open pattern, forming a "ring-like agglomeration and radiation" type. Resilience values are dispersed across most of the region. Resilient communities with middle and high-value human settlements are primarily concentrated in the central and southeastern parts of the old city. Conversely, northern, western, and southern communities demonstrate a balanced yet low level of resilience, showing a "flake-like agglomeration and radiation" spatial pattern. Overall, the central city mainly consists of low and medium resilience zones, with wider distribution. On the other hand, the peripheral areas are dominated by very low resilience zones, forming a semi-humped spatial pattern of "low inner circle, high middle circle and low outer circle".

Figure 11 displays the spatial distribution of the subsystems in the CRI model, indicating significant variation. The driving force index (D-system) shows a typical pyramidal spatial pattern. Higher values are found in the centre and lower values in the peripheral areas. The reason may be that Huangpu and Xuhui districts are the most economically developed areas in Shanghai. The development of enterprises not only increases the GDP of the region, but also attracts a large number of labors. The stress index (P-system) exhibits a patchy characteristic due to its dependence on district and county-level environmental pollution data. It displays a basin-type spatial pattern of "low in the center and high in the periphery," reflecting the overall seriousness of environmental pollution in the old city. The areas with high values of the stress index cover a wide area, up to three "red areas" and one "orange area ". The state index (S-system) generally maintains a low level of balanced distribution, with more high-value neighborhoods near the Huangpu River. The overall spatial pattern of "low in the west and high in the east" is formed. The reason for this may be the dense concentration of businesses and population along the river. This often requires more buildings and transport facilities. The impact index (I-system) showcases a "polar core" spatial pattern, with scattered high-value areas. Finally, the response index (R-system) appears as a "mosaic" type spatial pattern. The overall distribution is freely dispersed. But most of the high value areas are distributed in the west. The reason may be that the economic development level of this area is not so developed compared with Huangpu and Hongkou districts. There are more green spaces to be developed in the region.

### Driver factors analysis of community resilience for human settlement

The paper first calculates the standard deviation of the normalized indicator values. Then we use the geodetector technique to examine and analyze the impact on the resilience of human settlements.

### Factor detection analysis

This study employed the GIS-Jenks Natural Breaks Method to classify the original numerical quantities of the five variables, transforming them into categorical values based on the classification results. Then we introduced the CRI and categorical values into the respective dependent variable Y and independent variable X in the geographical detector to identify influential factors. A higher q-value indicates a stronger explanatory power of the independent variable X on the dependent variable Y.

The results in Table 7 demonstrate that R6 (distance from bus stops) holds the highest rank with a q-value of 0.292. It indicates that it is the most influential factor affecting the resilience of human settlements in the community. The proximity to public transportation reflects the infrastructure development of a city and represents an external response to its economic level. Therefore, future community development efforts should focus on enhancing public transport service facilities and innovations in transport technology.

**Table 7:** Results of CR level factor detection

	**D7**	**P1**	**S1**	**I1**	**R6**
q	0.12	0.12	0.11	0.077	0.292
p	＜0.001	＜0.001	＜0.001	＜0.001	＜0.001

D7 (average annual temperature) and P1 (CO_2_ emissions) both hold a q-value of 0.12, securing the second rank. This suggests that both D7 and P1 significantly impact community human settlement resilience. Sustainable urban development depends on the development of clean energy. Governments should increase the recycling rate of energy, reduce carbon dioxide emissions and mitigate the greenhouse effect.

S1 (building density) acquires a q-value of 0.11, ranking third. Although slightly lower than the second rank, this indicates that S1 has a notable influence on CR. It underscores the importance of building density as a crucial expression of community development.

I1 (number of jogging trajectories) holds the fifth rank with a q-value of 0.077, indicating a minor impact on CR. This suggests that human health activities have limited influence on human settlement resilience. However, it is important to note that human health activities cannot be dissociated from the environment and social support. Attention can be given to human health activities without compromising other aspects of community development.

### Interaction detection analysis

Interaction detection was employed to assess whether the combined effect of factors increased or decreased the explanatory power for the level of CR (Table 8). The results of factor interactions revealed that the values of the factor effects in two-way interactions were higher than those of single factors, demonstrating enhanced and nonlinear effects of the two-factor interactions. This indicates that the combined action of factors augmented the explanatory capacity of the resilience level. Specifically, the two-factor interactions between D7 and S1, D7 and I1, P1 and S1, P1 and I1, S1 and R6, and I1 and R6 exhibited significant effects on CR levels, indicating a robust association between these factors.

**Table 8:** Results of CR level interaction detection

Interaction	D7 ∩ P1	D7 ∩ S1	D7 ∩ I1	D7 ∩ R6	P1 ∩ S1	P1 ∩ I1	P1 ∩ R6	S1 ∩ I1	S1 ∩ R6	I1 ∩ R6
q-value	0.121	0.270^a^	0.202^a^	0.365	0.270^a^	0.202^a^	0.365	0.159	0.424^a^	0.375^a^

"^a^" is non-linear enhancement, i.e. D7 ∩ S1 > D7 + S1; the rest, not marked with a symbol, are two-factor enhancements, i.e. D7 + S1 > D7 ∩ S1 > D7,S1

### Correlation analysis

**Fig. 12:**
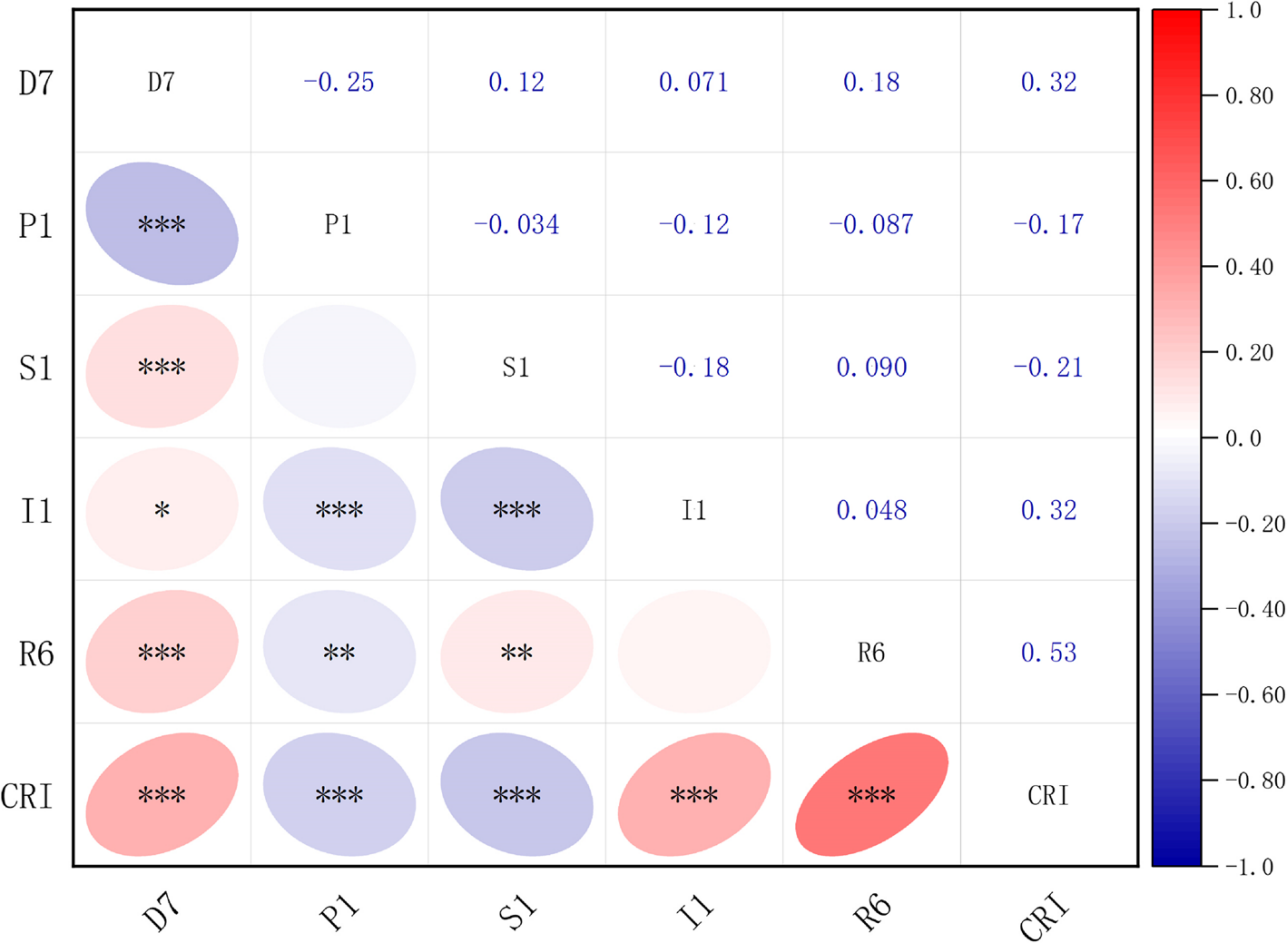
CRI correlation

As shown in Fig. 12, we analyzed the correlation between CRI and drivers using R language software and Origin software. The results indicate a significant positive correlation between the CRI and D7, I1, and R6, while exhibiting a negative correlation with P1 and S1. Notably, R6 exhibited the strongest influence on CR, with the highest correlation coefficient of 0.53 in absolute value. Both D7 and I1 displayed the same correlation strength with CR, with an absolute value of 0.32. On the other hand, P1 exhibited the weakest correlation with CR, with an absolute value of 0.17.

## Discussion

This study assessed the CRs in the study area based on the integrated evaluation index method of the DPSIR model. Also this study considered the interaction between health activities and environmental supply and demand.

The findings reveal that while some communities demonstrate excellent human settlement characteristics, well-developed infrastructure, and a strong capacity to withstand external disasters. However, the overall resilience of communities still needs to be improved. In general, most communities score lower in ecological environment, built environment, and social functions. Additionally, there is a significant disparity in human settlement resilience among communities, emphasizing the need for prompt improvement. This result can be attributed to severe urban growth. Urban growth has led to problems such as urban flooding, traffic congestion and ecological damage [81–83]. Consequently, this hinders the endogenous development momentum for human health activities [84, 85]. A positive trend in this element is crucial for enhancing the CR. Residents are the main contributors to community activities. If they lack healthy activities, it is difficult to maintain endogenous development dynamics. This trend is detrimental to the stable development of the community human settlement system [86, 87]. To enhance the resilience of the community human settlement system and achieve sustainable and high-quality development, it is necessary to adopt incentive response measures based on the current state of the system and its impact results. For instance, encouraging residents to engage in healthy physical activities and improving community sports facilities can bring positive feedback to the system.

The spatial distribution analysis reveals that at the district and county scale, CR follows a spatial pattern of being low in the center and high in the periphery. At the community scale, CR exhibits an irregular spatial pattern characterized by pole-core agglomeration and radiation. Communities with high resilience are predominantly found in the south-central and Huangpu River areas. The spatial patterns of the two types of systems vary considerably. This may be due to differences in the proportion of communities within the regional space. This leads to large differences in the comparison of resilience systems between districts and counties. The disparity in resilience between areas outside the polar nuclei and the polar nuclei can be attributed to the daily health activities of urban residents being concentrated and dispersed mainly in the monocentric clustering of polar nuclei in the district centers. The spatial clustering of resilience is moderately distributed in most areas. This reflects a spatial supply–demand mismatch or complementary relationship between health activities and the environment. This leads to a low-level equilibrium in the spatial distribution characteristics of CR, aligning with existing research [88–90]. Furthermore, it is observed that high resilience areas are primarily concentrated in the central and southeastern parts of Shanghai's old city, including Huangpu District, Xuhui District, and Jing'an District. These areas serve as the core regions for integrated urban functions, characterized by intensive and frequent daily health activities. On the other hand, the northern, northeastern, and western areas of the old city, and the southern end of the city exhibit weaker resilience capacity due to imperfect integrated functions and location levels of the activity environment. These areas also display less dynamism in terms of health activity [91]. To overcome the spatial layout inertia of one-way overdraft in the "center-periphery" pattern, efforts should focus on promoting the orderly distribution of resilient elements related to urban residents' health activities and environmental systems. This requires ensuring that spaces for everyday health activities are autonomous, balanced and inclusive, rather than relying solely on static differences in spatial hierarchical scales and functional positioning.

In addition, based on the results of the spatial distribution of subsystems, we recommend the following actions. Firstly, there is a need to accelerate the orderly decentralization and relocation of over-concentrated public service resources and population from the old city center to peripheral communities. This will promote comprehensive development, improved supporting facilities, and population concentration in the new central area, enhancing the concentration of modern and traditional living atmospheres. Secondly, the government should vigorously promote the construction of an integrated slow-moving transport network and the promotion of mixed use of various land uses among key communities. This can link health activities and environmental elements through multiple channels. This facilitates the micro-circulation of health activities within communities, forming balanced spatial clusters for daily health activities. In addition, it is necessary to promote the organic regeneration of older urban neighborhoods and the planned development of new towns. This enhances synergies and complementarities between communities in the region. This reduces the gradient among communities and optimizes two-way interaction, enhancing the efficiency, effectiveness, and quality of daily health activities.

Regarding the contribution results, it is evident that the construction of community public transport services can promote environmental and economic development, which constitutes the primary reason for the increase in CR in the study area [82, 93]. Reducing the distance between the community and public transport stations can further enhance CR. The average annual temperature and CO_2_ emissions also exert a significant impact on CR. Therefore, natural environmental stresses such as climate, air pollution and vegetation are key factors influencing changes in CR. Building density and the number of jogging trajectories serve as indicators of the vitality of human activity systems in the face of environmental disturbances. Communities with low building density and dense jogging trajectories demonstrate higher resilience. Furthermore, it is worth noting that the correlation between annual mean temperature, jogging activity, and CR exhibits a significantly positive and non-linearly increasing relationship. This suggests that human health activities are influenced by temperature, resulting in notable changes in resilience. Conversely, the non-linear increase in CO_2_ and building density signifies an intensification of the greenhouse effect and severe urban sprawl due to human activities related to urbanization. These factors contribute to an overall low level of CR. Therefore, it is strongly recommended that governments worldwide focus on the rational use of ecological resources and control the extent of urban community growth during rapid urbanization. Simultaneously, efforts should be made to strengthen the protection of existing woodland and grassland vegetation, especially by promoting residents' engagement in health activities. These measures ensure the sustainability of community human settlements.

### Advantages and limitations

The study of the resilience of human settlements is of great importance in the field of global geographic studies. While urban human settlements and urban resilience have gained attention, there is still a scarcity of academic research specifically focused on human settlement resilience. This paper incorporates the principle of resilience into the study of human settlements and addresses the spatial supply–demand contradiction related to daily health activities as a primary community conflict. By exploring the dynamic relationship between people and land, the study reveals the human values and micro-details of spatiotemporal interactions. And the paper establishes an organically integrated CR evaluation index system through comprehensive correlation and multiple characterization of resilience elements and capacities. This research contributes to the academic understanding of resilience.

The research methodology employed in this paper is reasonable. Based on the DPSIR model, this paper analyses the interactions between the elements from a system perspective. This paper further constructs an evaluation index system for urban habitat resilience. Previous studies have used various methods to investigate the influencing mechanisms of geographic environmental phenomena, but they often lack the identification of interactions among multiple variables. In contrast, this study utilizes geodetector technology to explore the driving forces that impact the level of CR. This approach effectively identifies relationships among multiple variables, leading to a better understanding of the mechanisms at play.

The feasibility of the research data is supported by geographic big data, which enables comprehensive assessment and spatial visualization of urban resilience. This solid foundation facilitates scientific governance practices and spatial carriers for urban resilience. The study offers valuable insights for expanding the research field, deepening theoretical understanding, improving measurement methods, and enhancing CR governance.

However, there are limitations in this paper. The community human settlement system is a complex and open system, and solely understanding the five major subsystems is insufficient. It is necessary to examine the interactions among various subsystems and enhance the knowledge of overall CR. Additionally, due to data acquisition limitations, this paper has selected relatively important and representative indicators. Further research should refine data acquisition and processing methods and improve the indicator system for measuring the CR. Furthermore, since community human settlement systems are constantly evolving, it is important to track their evolution over time and adjust the research methodology based on macroscopic observations. This will allow for continuous follow-up research on the resilience of community human settlement systems.

## Conclusion

In the complex context of global environmental change and urbanization, the dynamic and evolving contradiction between the supply and demand for healthy living necessitates the promotion of a more resilient supply and demand process. This paper first constructs a CR evaluation index system based on the DPSIR model. Then this paper conducted a comprehensive evaluation of the resilience of Shanghai central habitat by integrating the AHP-entropy method, GIS spatial analysis and geographical detector method. The main findings of the study are as follows:

1. There are significant variations in the level of human settlement resilience across the study area, exhibiting a basic "slide-shaped" fluctuation tendency.
2. The spatial distribution of human settlement resilience shows two patterns. One is the " pole-core agglomeration radiation" type characterized by core colonies with very high resilience values. The other is the typical irregular, open "ring agglomeration radiation" type dominated by core colonies with medium and high resilience values.
3. Geographical detection, interaction detection, and correlation analysis highlight the dominant factors influencing CR. The analysis indicates that the CRI is positively correlated with average annual temperature and the number of jogging trajectories, while negatively correlated with CO_2_ emissions and building density. In addition, the interactions between these factors enhanced resilience in a non-linear and bivariate manner.

The results of this study hold significant implications for countries and communities worldwide. Firstly, the findings emphasize the importance of evaluating CR, particularly about health activities and the balance between environmental supply and demand. This highlights the need for governments and communities to prioritize CR and enhance the quality of life and health of community residents. Secondly, the comprehensive evaluation methods and techniques employed in this study can serve as a reference to develop their evaluation indicator systems and methods suitable for local community human settlements. Finally, this study provides insights for countries to formulate relevant environmental policies and climate change adaptation strategies, so as to safeguard human health and promote environmentally sustainable development.

## Data Availability

The data that support the findings of this study are available from the Dorray Sports app. Restrictions apply to the availability of these data, which were used under license for this study.

## Abbreviations

CR: Community human settlement resilience
CRI: Community human settlement resilience index
HH: High-High
LL: Low-Low
HL: High-Low
LH: Low–High

## References

[1] Ma RF, Wang TF, Zhang WZ, Yu JH, Wang D, Chen L, Jiang YP, Feng GQ (2016). Overview and progress of Chinese geographical human settlement research. J Geog Sci.

[2] Wu F, Liu Y, Zeng YY, Yan H, Zhang Y, Li LH (2020). Evaluation of the human settlements environment of public housing community: a case study of Guangzhou. Sustainability.

[3] Qiu BK, Li HL, Zhou M, Zhang L (2015). Vulnerability of ecosystem services provisioning to urbanization: A case of China. Ecol Ind.

[4] Williams DS, Costa MM, Sutherland C, Celliers L, Scheffran J (2019). Vulnerability of informal settlements in the context of rapid urbanization and climate change. Environ Urban.

[5] Liu L, Luo Y, Pei JJ, Wang HQ, Li JX, Li Y (2021). Temporal and spatial differentiation in urban resilience and its influencing factors in Henan Province. Sustainability.

[6] Zhang H, Liang XY, Chen H, Shi QQ. Spatio-temporal evolution of the social-ecological landscape resilience and management zoning in the loess hill and gully region of China. Environ Dev. 2021;39:100616.

[7] Cheng T, Zhao YH, Zhao CJ. Exploring the spatio-temporal evolution of economic resilience in Chinese cities during the COVID-19 crisis. Sustain Cities Soc. 2022;84:103997.

[8] He D, Miao PJ, Qureshi NA. Can industrial diversification help strengthen regional economic resilience? Front Environ Sci. 2022;10:987396.

[9] Yang M, Jiao MY, Zhang JY (2022). Spatio-temporal analysis and influencing factors of rural resilience from the perspective of sustainable rural development. Int J Environ Res Public Health.

[10] Gillespie-Marthaler L, Nelson K, Baroud H, Abkowitz M (2019). Selecting Indicators for Assessing Community Sustainable Resilience. Risk Anal.

[11] Abdul-Rahman M, Soyinka O, Adenle YA, Chan EHW. Comparative study of the critical success factors (CSFs) for community resilience assessment (CRA) in developed and developing countries. Int J Disaster Risk Reduct. 2022;77:103060.

[12] Auliagisni W, Wilkinson S, Elkharboutly M (2022). Learning from floods-how a community develops future resilience. Water.

[13] Song JL, Pandey R, Dong GP, Sharifi A, Subedi BP. Urban-Rural Disparity in Community Resilience: A Multilevel Analysis of the Relief Progress after the (2015). Nepal Earthquake. Sustain Cities Soc.

[14] Seidl R, Spies TA, Peterson DL, Stephens SL, Hicke JA (2016). Searching for resilience: addressing the impacts of changing disturbance regimes on forest ecosystem services. J Appl Ecol.

[15] Schirpke U, Kohler M, Leitinger G, Fontana V, Tasser E, Tappeiner U (2017). Future impacts of changing land-use and climate on ecosystem services of mountain grassland and their resilience. Ecosyst Serv.

[16] Chambers JC, Allen CR, Cushman SA (2019). Operationalizing ecological resilience concepts for managing species and ecosystems at risk. Front Ecol Evol.

[17] Zhao RD, Fang CL, Liu HM, Liu XX. Evaluating urban ecosystem resilience using the DPSIR framework and the ENA model: a case study of 35 cities in China. Sustain Cities Soc. 2021;72:102997.

[18] Tang JH, Xiong KN, Chen Y, Wang Q, Ying B, Zhou JY (2022). A review of village ecosystem vulnerability and resilience: implications for the rocky desertification control. Int J Environ Res Public Health.

[19] Sarkki S, Ficko A, Wielgolaski FE, Abraham EM, Bratanova-Doncheva S, Grunewald K, Hofgaard A, Holtmeier FK, Kyriazopoulos AP, Broll G (2017). Assessing the resilient provision of ecosystem services by social-ecological systems: introduction and theory. Climate Res.

[20] Stori FT, Peres CM, Turra A, Pressey RL (2019). Traditional ecological knowledge supports ecosystem-based management in disturbed coastal marine social-ecological systems. Front Mar Sci.

[21] Shahidullah AKM, Choudhury MU, Haque CE (2020). Ecosystem changes and community wellbeing: social-ecological innovations in enhancing resilience of wetlands communities in Bangladesh. Local Environ.

[22] Tai XL, Xiao W, Tang YX. A quantitative assessment of vulnerability using social-economic-natural compound ecosystem framework in coal mining cities. J Clean Prod. 2020;258:120969.

[23] Stotten R, Ambrosi L, Tasser E, Leitinger G (2021). Social-ecological resilience in remote mountain communities: toward a novel framework for an interdisciplinary investigation. Ecol Soc.

[24] de Bruijn K, Buurman J, Mens M, Dahm R, Klijn F (2017). Resilience in practice: Five principles to enable societies to cope with extreme weather events. Environ Sci Policy.

[25] Xue XL, Wang L, Yang RJ (2018). Exploring the science of resilience: critical review and bibliometric analysis. Nat Hazards.

[26] Feng XH, Xiu CL, Bai LM, Zhong YX, Wei Y. Comprehensive evaluation of urban resilience based on the perspective of landscape pattern: a case study of Shenyang city. Cities. 2020;104:102722.

[27] Wu XL, Hu F. Analysis of ecological carrying capacity using a fuzzy comprehensive evaluation method. Ecol Indic. 2020;113:106243.

[28] Lu Y, Li R, Mao XI, Wang SH. Towards comprehensive regional resilience evaluation, resistance, recovery, and creativity: From the perspective of the (2008). Wenchuan Earthquake. International Journal of Disaster Risk Reduction.

[29] Zhao RD, Fang CL, Liu J, Zhang LF. The evaluation and obstacle analysis of urban resilience from the multidimensional perspective in Chinese cities. Sustain Cities Soc. 2022;86:104160.

[30] Huang GY, Li DZ, Zhu XW, Zhu J. Influencing factors and their influencing mechanisms on urban resilience in China. Sustain Cities Soc. 2021;74:103210.

[31] Jolgehnejad AK, Kahnali RA, Heyrani A (2021). Factors Influencing Hospital Resilience. Disaster Med Public Health Prep.

[32] Lin YZ, Peng C, Shu JF, Zhai W, Cheng JQ (2022). Spatiotemporal characteristics and influencing factors of urban resilience efficiency in the Yangtze River Economic Belt. China Environmental Science and Pollution Research.

[33] Cacioppo JT, Reis HT, Zautra AJ (2011). Social Resilience The Value of Social Fitness With an Application to the Military. Am Psychol.

[34] Quinlan AE, Berbes-Blazquez M, Haider LJ, Peterson GD (2016). Measuring and assessing resilience: broadening understanding through multiple disciplinary perspectives. J Appl Ecol.

[35] Wister A, Klasa K, Linkov I. A Unified Model of Resilience and Aging: Applications to COVID-19. Frontiers in Public Health. 2022; 10. 10.3389/fpubh.2022.865459 PMC9170899 35685765

[36] Bartos SE, Langdridge D (2019). LGBQ resilience: a thematic meta-synthesis of qualitative research. Psychology & Sexuality.

[37] Wilson CA, Walker D, Saklofske DH (2021). Developing a model of resilience in older adulthood: a qualitative meta-synthesis. Ageing Soc.

[38] Ang WHD, Shorey S, Lopez V, Chew HSJ, Lau Y (2022). Generation Z undergraduate students' resilience during the COVID-19 pandemic: a qualitative study. Curr Psychol.

[39] Wu Y, Que W, Liu YG, Cao L, Liu SB, Zhang J. Is resilience capacity index of Chinese region performing well? Evidence from 26 provinces. Ecol Indic. 2020;112:106088.

[40] Wang ZY, Chen ZQ, Ma CP, Wennersten R, Sun Q (2022). Nationwide evaluation of urban energy system resilience in China using a comprehensive index method. Sustainability.

[41] Chuang WC, Garmestani A, Eason TN, Spanbauer TL, Fried-Petersen HB, Roberts CP, Sundstrom SM, Burnett JL, Angeler DG, Chaffin BC (2018). Enhancing quantitative approaches for assessing community resilience. J Environ Manage.

[42] Suna RR, Shia SH, Rehemana YJ, Lib SM. Measurement of urban flood resilience using a quantitative model based on the correlation of vulnerability and resilience. Int J Disaster Risk Reduct. 2022;82:103344.

[43] Dur M, Steiner G, Stoffer MA, Fialka-Moser V, Kautzky-Willer A, Dejaco C, Ekmekcioglu C, Prodinger B, Binder A, Smolen J (2016). Initial evidence for the link between activities and health: Associations between a balance of activities, functioning and serum levels of cytokines and C-reactive protein. Psychoneuroendocrinology.

[44] Nieuwendyk LM, Belon AP, Vallianatos H, Raine KD, Schopflocher D, Spence JC, Plotnikoff RC, Nykiforuk CI (2016). How perceptions of community environment influence health behaviours: using the Analysis Grid for Environments Linked to Obesity Framework as a mechanism for exploration. Health Promotion and Chronic Disease Prevention in Canada-Research Policy and Practice.

[45] Hu QY, Wang C. Quality evaluation and division of regional types of rural human settlements in China. Habitat Int. 2020;105:102278.

[46] Liu H, Li XM (2022). Understanding the driving factors for urban human settlement vitality at street level: a case study of Dalian, China. Land.

[47] Tian SZ, Qi A, Li ZH, Pan XB, Liu YS, Li XM (2022). Urban "Three States" human settlements high-quality coordinated development. Buildings.

[48] Wang HF, Chiou SC (2019). Study on the sustainable development of human settlement space environment in traditional villages. Sustainability.

[49] Cong XP, Li XM, Gong YL (2021). Spatiotemporal evolution and driving forces of sustainable development of urban human settlements in China for SDGs. Land.

[50] Guan YY, Li XM, Yang J, Li SB, Tian SZ (2022). Spatial differentiation of comprehensive suitability of urban human settlements based on GIS: a case study of Liaoning Province. China Environment Development and Sustainability.

[51] Xie TC, Liu XY, Nie PJ (2022). Study on spatial-temporal patterns and factors influencing human settlement quality in Beijing. Sustainability.

[52] Zhu LK, Guo YY, Zhang C, Meng JJ, Ju L, Zhang YS (2020). Assessing community-level livability using combined remote sensing and internet-based big geospatial data. Remote Sens.

[53] Wang Y, Miao ZY (2022). Towards the analysis of urban livability in China: spatial-temporal changes, regional types, and influencing factors. Environ Sci Pollut Res.

[54] Sanyal J, Lu XX (2005). Remote sensing and GIS-based flood vulnerability assessment of human settlements: a case study of Gangetic West Bengal. India Hydrological Processes.

[55] Chen JS (2022). Temporal-spatial assessment of the vulnerability of human settlements in urban agglomerations in China. Environ Sci Pollut Res.

[56] Chen B, Chen YJ, Chen Y, Gao J (2022). Model of demand of human settlement environment for rural houses in North China: a structural equation modeling approach. Buildings.

[57] Ye L, Wu ZH, Wang T, Ding KL, Chen Y (2022). Villagers' satisfaction evaluation system of rural human settlement construction: empirical study of Suzhou in China's rapid urbanization area. Int J Environ Res Public Health.

[58] Li XM, Liu H (2021). The influence of subjective and objective characteristics of urban human settlements on residents' life satisfaction in China. Land.

[59] Li YC, Liu CX, Zhang H, Gao X (2011). Evaluation on the human settlements environment suitability in the Three Gorges Reservoir Area of Chongqing based on RS and GIS. J Geog Sci.

[60] Li Y, Zhang XT, Gao XX (2022). An evaluation of the coupling coordination degree of an urban economy-society-environment system based on a multi-scenario analysis: the case of Chengde City in China. Sustainability.

[61] Yu X, Chen HX. Study on coupling coordination of the human settlement environment and tourism industry in the yellow river basin. Front Environ Sci. 2022;10:1016839.

[62] Ge DD, Zheng YY, Zhang SN, Fu JY, Su F (2022). Spatio-temporal pattern and influence mechanism of rural human settlements system resilience: case from China. Sustainability.

[63] Ladi T, Mahmoudpour A, Sharifi A (2022). Assessing environmental impacts of transportation sector by integrating DPSIR framework and X-Matrix. Case Studies on Transport Policy.

[64] Duan TT, Feng JS, Zhou YQ, Chang X, Li YX. Systematic evaluation of management measure effects on the water environment based on the DPSIR-Tapio decoupling model: a case study in the Chaohu Lake watershed, China. Sci Total Environ. 2021;801:149528. 10.1016/j.scitotenv.2021.149528 34418629

[65] Chuai XM, Fan C, Wang MS, Wang JJ, Han YJ (2020). A study of the socioeconomic forces driving air pollution based on a DPSIR model in Henan Province, China. Sustainability.

[66] Wei YG, Zhu XH, Li Y, Yao T, Tao Y (2019). Influential factors of national and regional CO2 emission in China based on combined model of DPSIR and PLS-SEM. J Clean Prod.

[67] Lu JJ, Liang LC, Feng Y, Li RN, Liu Y (2015). Air Pollution Exposure and Physical Activity in China: Current Knowledge, Public Health Implications, and Future Research Needs. Int J Environ Res Public Health.

[68] Yousafzai S, Saeed R, Rahman G, Farish S (2022). Spatio-temporal assessment of land use dynamics and urbanization: linking with environmental aspects and DPSIR framework approach. Environ Sci Pollut Res.

[69] Gu ZN, Luo XL, Chen YR, Liu XM, Xiao CR, Liang YF (2022). Density, diversity, and design: evaluating the equity of the elderly communities in three measures of the built environment. Land.

[70] Xu Z, Coors V (2012). Combining system dynamics model, GIS and 3D visualization in sustainability assessment of urban residential development. Build Environ.

[71] Wang ZY, Qin ZZ, He J, Ma YY, Ye Q, Xiong YQ (2019). The association between residential density and physical activity among urban adults in regional China. BMC Public Health.

[72] Carver A, Timperio A, Hesketh K, Crawford D (2010). Are Safety-Related Features of the Road Environment Associated with Smaller Declines in Physical Activity among Youth?. Journal of Urban Health-Bulletin of the New York Academy of Medicine.

[73] Kavanagh SA, Hawe P, Shiell A, Mallman M, Garvey K (2022). Soft infrastructure: the critical community-level resources reportedly needed for program success. BMC Public Health.

[74] McMahon SK, Park YS, Lewis B, Guan WH, Oakes JM, Wyman JF, Rothman AJ (2019). Older Adults' Utilization of Community Resources Targeting Fall Prevention and Physical Activity. Gerontologist.

[75] Drieling RL, Rosas LG, Ma J, Stafford RS (2014). Community Resource Utilization, Psychosocial Health, and Sociodemographic Factors Associated with Diet and Physical Activity among Low-Income Obese Latino Immigrants. J Acad Nutr Diet.

[76] Stanesby O, Morse M, Magill L, Ball K, Blizzard L, Harpur S, et al. Characteristics associated with willingness to walk further than necessary to the bus stop: insights for public transport-related physical activity. J Transp Health. 2021;22:101139.

[77] de Jalon SG, Chiabai A, Quiroga S, Suarez C, Scasny M, Maca V, et al. The influence of urban greenspaces on people's physical activity: a population-based study in Spain. Landsc Urban Plan. 2021;215:104229.

[78] Villeneuve PJ, Ysseldyk RL, Root A, Ambrose S, DiMuzio J, Kumar N (2018). Comparing the normalized difference vegetation index with the Google street view measure of vegetation to assess associations between greenness, walkability, recreational physical activity, and health in Ottawa, Canada. Int J Environ Res Public Health.

[79] Cao F, Ge Y, Wang JF (2013). Optimal discretization for geographical detectors-based risk assessment. Giscience & Remote Sensing.

[80] Zhang ZH, Song YZ, Wu P. Robust geographical detector. Int J Appl Earth Obs Geoinf. 2022;109:102782.

[81] Huang ZH, Du XJ. Urban land expansion and air pollution: evidence from China. J Urban Plan Dev. 2018;144(4):05018017.

[82] Wang YX, Zhu YX, Li XS, Cai AN, Wang XY, Zhang C (2022). Spatiotemporal variation of urban thermal environment and its relationship with urban expansion types from 2000 to 2020: a case of Huai'an central urban area, Huai'an. China Geomatics Natural Hazards & Risk.

[83] Zhao MY, Cai HY, Qiao Z, Xu XL (2016). Influence of urban expansion on the urban heat island effect in Shanghai. Int J Geogr Inf Sci.

[84] Shi G, Shan J, Ding L, Ye P, Li Y, Jiang N (2019). Urban road network expansion and its driving variables: a case study of Nanjing City. Int J Environ Res Public Health.

[85] Zhong JJ, Liu WT, Niu BQ, Lin XB (2022). Deng YH.

[86] Reams MA, Irving JK (2019). Applying community resilience theory to engagement with residents facing cumulative environmental exposure risks: lessons from Louisiana's industrial corridor. Rev Environ Health.

[87] Treichler EBH, Glorioso D, Lee EE, Wu TC, Tu XM, Daly R, O'Brien C, Smith JL, Jeste DV (2020). A pragmatic trial of a group intervention in senior housing communities to increase resilience. Int Psychogeriatr.

[88] Jiang YP, Sun HH (2021). Exploring the characteristics and influencing factors of leisure walking based on the demand of behavior. Sustainability.

[89] Schrammeijer EA, Malek Z, Verburg PH. Mapping demand and supply of functional niches of urban green space. Ecol Indic. 2022;140:109031.

[90] Xu X, Hu J, Lv L, Yin JJ, Tian XB (2022). Research on the matching relationship between the supply of urban ecological recreational space and the demand of residents-a case study of an urban development area in Wuhan. Int J Environ Res Public Health.

[91] Chen TT, Hui ECM, Lang W, Tao L (2016). People, recreational facility and physical activity: New-type urbanization planning for the healthy communities in China. Habitat Int.

[92] Han I, Samarneh L, Stock TH, Symanski E (2018). Impact of transient truck and train traffic on ambient air and noise levels in underserved communities. Transportation Research Part D-Transport and Environment.

[93] Anciaes P, Jones P, Mindell JS, Scholes S (2022). The cost of the wider impacts of road traffic on local communities: 1.6% of Great Britain's GDP. Transportation Research Part a-Policy and Practice.

